# A Numerical Study of Local Variations in Tidal Regime of Tagus Estuary, Portugal

**DOI:** 10.1371/journal.pone.0080450

**Published:** 2013-12-02

**Authors:** João Miguel Dias, Juliana Marques Valentim, Magda Catarina Sousa

**Affiliations:** CESAM, Departamento de Física, Universidade de Aveiro, Campus de Santiago, Aveiro, Portugal; University of Vigo, Spain

## Abstract

Tidal dynamics of shallow estuaries and lagoons is a complex matter that has attracted the attention of a large number of researchers over the last few decades. The main purpose of the present work is to study the intricate tidal dynamics of the Tagus estuary, which states as the largest estuary of the Iberian Peninsula and one of the most important wetlands in Portugal and Europe. Tagus has large areas of low depth and a remarkable geomorphology, both determining the complex propagation of tidal waves along the estuary of unknown manner. A non-linear two-dimensional vertically integrated hydrodynamic model was considered to be adequate to simulate its hydrodynamics and an application developed from the SIMSYS2D model was applied to study the tidal propagation along the estuary. The implementation and calibration of this model revealed its accuracy to predict tidal properties along the entire system. Several model runs enabled the analysis of the local variations in tidal dynamics, through the interpretation of amplitude and phase patterns of the main tidal constituents, tidal asymmetry, tidal ellipses, form factor and tidal dissipation. Results show that Tagus estuary tidal dynamics is extremely dependent on an estuarine resonance mode for the semi-diurnal constituents that induce important tidal characteristics. Besides, the estuarine coastline features and topography determines the changes in tidal propagation along the estuary, which therefore result essentially from a balance between convergence/divergence and friction and advection effects, besides the resonance effects.

## Introduction

**Figure 1:**
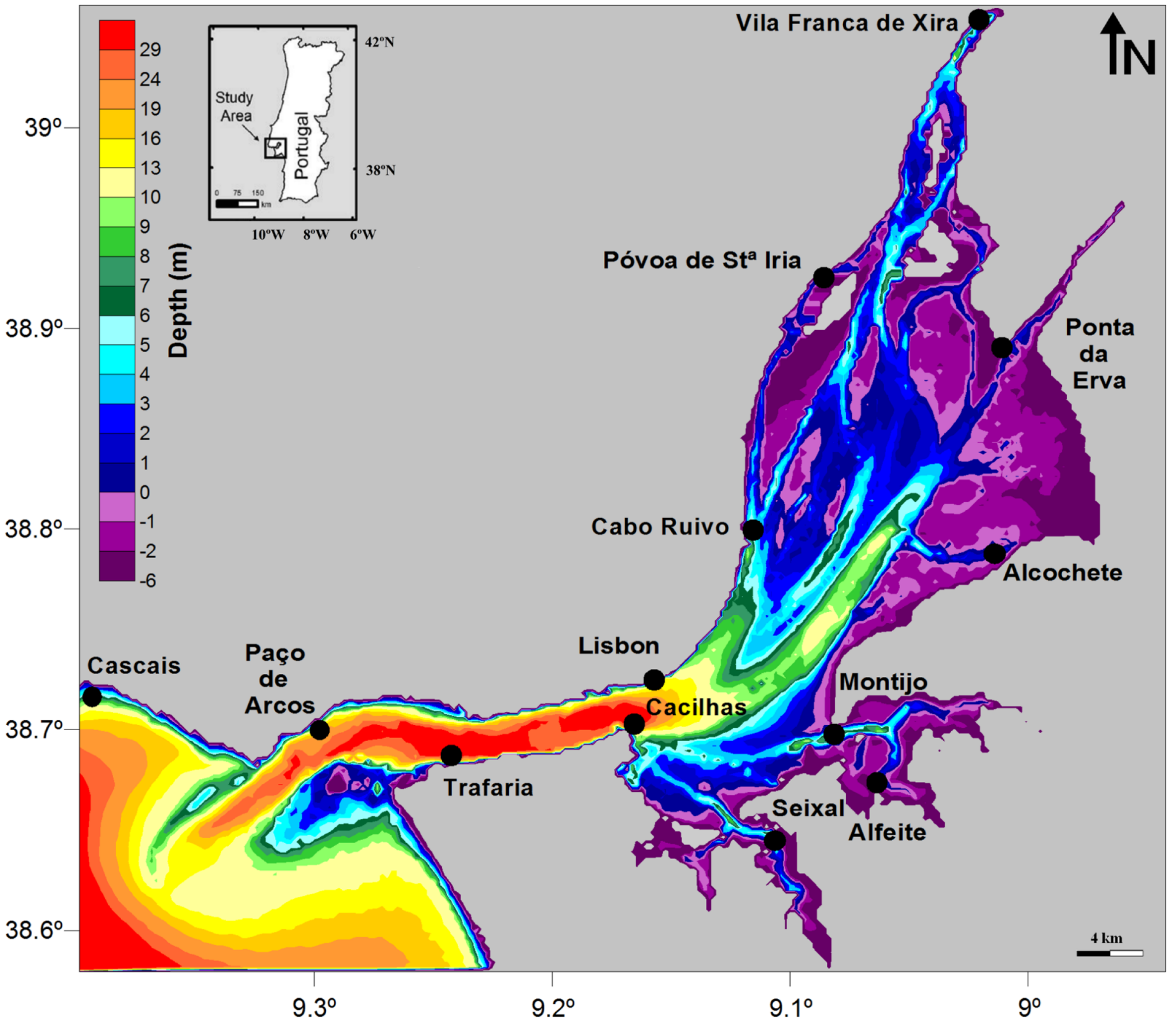
Geographic location of Tagus estuary and the numerical bathymetry, with depth in meters relative to the chart datum (2.0 m below the mean sea level) and with locations of the stations used in the numerical model calibration.

The water movements and the turbulent mixture that result from the tidal forcing express problems and interesting challenges in the hydrodynamic field. Knowledge of tidal heights and tidal currents structure is therefore essential to understand problems such as dispersion rate of pollutants, sediment transport and erosion processes in coastal areas [1]. Moreover, tidal asymmetries strongly influence nutrient balances, sediment loads, particles and pollutants transportations, etc. [2]. Thus, the understanding of the central processes lined by the tidal wave propagation seems to be crucial to obtain an overview concerning to the different uses of the coastal systems and in this particular study, of the Tagus estuary (Fig. 1), which dynamics is mainly ruled by the tidal forcing [3].

This coastal system is one of the largest estuaries of the West coast of Europe and is located in the most populated area of Portugal, including the capital Lisboa [4]. Likewise, Tagus estuary represents an important ecological system that is classified as a natural reserve since 1976 (Ramsar Convention on Wetlands). However, most of its margins are occupied by anthropogenic activities [5], and consequently is still subject to considerable urban and industrial pressures that induce several environmental problems including contamination by heavy metals and faecal material. In fact, this estuary plays an important role at a metropolitan and national level, due to two main aspects: it has a central environmental element in the Lisboa Metropolitan Area, which arises from its biological potential and its natural and unique heritage richness; it concentrates several uses and economic and social functions – tourism, recreation, leisure, industry, agriculture, aquaculture, ports, urban areas – which need to be in balance with development [6]. Nevertheless, both the ecological and human values may be affected by near future sea level rise (SLR) [5].

[7], for example, in a study about SLR impacts in Tagus estuary salt marshes dynamics, indicates that instantaneous velocity intensity will increase in a climate change scenario, promoting a rising tendency for the sediments exportation outwards the system, which represent a serious threat for the stability of the system margins and consequently for the surrounding ecosystems. These expected hydrodynamic changes will increase margins instability and contribute to coastal erosion. Furthermore [8], in a study about the vulnerability of the Tagus estuary margins, indicate an increasing vulnerability of urban areas to SLR, being important the knowledge of the present system patterns to develop future planning, management guidelines and adaptive measures to SLR. Deeply studies about SLR effect in Tagus estuary hydrodynamics will be crucial to develop strategies to minimize climate change impacts. To support such studies, it is essential to understand the current hydrodynamic features and patterns of the Tagus estuary.

However, numerous studies have been performed concerning the biogeochemical dynamics of this estuary ([9], [10], [11], [12], [13] and [14]) while despite its relevance in the overall estuarine dynamics, the amount of work dedicated to its physical dynamics, and especially to its tidal properties, is limited. The contributions found in this field are in fact restricted. [15] and [16] performed a numerical study considering the 3-dimensional currents dynamics at the estuary mouth and the effect of tidal flats on estuary hydrodynamics. Recently [3], described the main forcing mechanisms role on the estuarine dynamics, finding that tide is the estuary key forcing mechanism, although river discharge influences the water column stratification in a seasonal time scale. The meteorological effects (wind and air temperature) are essentially seasonal, influencing the surface water temperature [17]. investigated the Tagus estuary plume dispersion induced by wind and river flow forcing using a 3-dimensional nested models set, concluding that the plume dynamic besides the importance of the freshwater discharge is highly influenced by the coastline geography and by the wind regime [18]. performed a preliminary study of Tagus estuary tidal dynamics through an analysis of the amplitude, phase and tidal ellipses parameters of the main semi-diurnal and diurnal tidal constituents, revealing that they are extremely dependent on the estuarine topography and coastline features.

This work aims to provide a deepest analysis of local variations of the Tagus estuary tidal dynamics, also giving support to ongoing biogeochemical research. More precisely, this work aims to deepen the previous study by [18], verifying the relation between the estuary dynamics and its topography and coastline characteristics, thus presenting further details and actual information about the Tagus estuary hydrodynamic features. The hydrodynamic model setup and calibration is also described in order to demonstrate the accuracy of this modelling application to study and understand the Tagus tidal dynamics. For that, a numerical modelling study of the tidal wave propagation along the estuary was performed, seeking to determine and to analyse the amplitude and phase patterns of the main tidal constituents, the tidal asymmetry, the tidal ellipses, the form factor and the tidal dissipation. Besides the Tagus dynamics knowledge improvement, these results will also contribute for a deepest understanding of the biogeochemical processes in this system.

## Study Area

Covering an area of approximately 320 km^2^ and with a mean volume of 188 km^3^, the Tagus estuary is one of the largest estuaries of Europe and it is the most extensive wetland area of the Portuguese territory ([7] and [19]). Its width varies between 400 m at its head and 15 km in the central bay and has an average depth of 5.1 m [16].

Morphologically, Tagus can be divided in two distinct regions that present different morphologies and properties: the lower and upper estuary (Fig. 1). The lower estuary is a channel of about 30 m depth that connects with the Atlantic Ocean and opens in a large bay (upper estuary) on the east side. The upper estuary (bay), widest and shallowest, extends from Vila Franca de Xira to the main channel (lower estuary) and it is characterized by extensive zones of tidal flats, salt marshes, small islands and a net of narrow channels [7] and [15].

The Tagus river is the major estuarine source of freshwater, with an annual average flow of ∼370 m^3^/s [3]. Other freshwater inputs, the Sorraia and the Trancão rivers, are comparatively small, with average annual discharges of ∼35 and ∼2.5 m^3^/s, respectively [3]. According to [3], the river discharge forcing mechanism acts essentially on a seasonal time scale, mainly influencing the surface layer salinity. In fact, Tagus has a mean tidal prism of ∼60×10^7^ m^3^ and the river inflow per tidal cycle is ∼8.2×10^6^ m^3^ [3], which means that the freshwater input is only about 1.4% of the marine water input during the same period. The estuary should be thus vertically homogeneous during large periods and dominated by tidal forcing.

The Tagus is ebb dominated with floods typically one hour longer than ebbs (stronger velocities during ebbs), and thus inducing a net export of sediments [16]. The area affected by tides reaches 80 km landward of Lisboa and maximum tidal currents achieve ∼2.0 m/s [12]. This mesotidal estuary presents an average tidal range at the seaward end of 2.4 m, and an extreme range changing from 0.9 m in neap tide to 4.1 m in spring tide. Tides are primary semi-diurnal and the tide form number is equal to 0.10 in Cascais (below the limit for mixing tides) [15]. *M_2_* is the dominant tidal constituent with amplitudes of ∼1 m. The phase difference between Cascais and Vila Franca de Xira is ∼1 h20 min for semi-diurnal constituents [16]. According to [16], the amplitudes of astronomic constituents grow rapidly in the lower estuary and more steadily in the upper estuary, up to Póvoa Sta. Iria, and then decrease up to Vila Franca de Xira. The shallow water constituents are negligible in the coastal region, but increase rapidly inside the system [16].

## Hydrodynamic Model

Previous hydrodynamic modelling implementations for the Tagus estuary in general have considered barotropic models, since the estuary is well mixed or partially mixed, except in flood scenarios [3]. Therefore, in this work, a modified version of SIMSYS2D ([20], [21] and [22]), based on the shallow water equations is used, expressing the mass and momentum conservation, and assuming incompressibility and hydrostatic pressure. In order to describe the relevant areas of tidal flats in this estuary, the model grid cells are allowed to dry/flood in a complex process described in [20]. This model was recently used with success in the hydrodynamic study of several coastal areas, as: Ria de Aveiro lagoon (Portugal) [22], [23], [24], [25], [26] and [27]; Lima estuary (Portugal) [28] and [29]; Tagus estuary (Portugal) [18], Patos lagoon (Brazil) [30] and Maputo bay (Mozambique) [31].

In this particular case, before applying this model it is essential to describe the hydrodynamic model setup and calibration for Tagus estuary, in order to demonstrate the accuracy of this modelling application to study and understand the Tagus tidal dynamics.

### Model setup

The major limitation of previous modelling applications developed for Tagus estuary has been the low horizontal resolution of the grids used, insufficient to properly resolve all channels [32]. In this work we chose to develop a grid with a resolution higher than that used in previous studies, considering the dimensions Δ*x* = Δ*y* = 200 m. The numerical bathymetry (Fig. 1) was developed from topo-hydrographical data measured by the Hydrographical Institute of the Portuguese Navy. A Krigging method was used to generate the rectangular computational grid, resulting in 227 cells in the *x* direction and 202 cells in the *y* direction.

In order to optimize the computational time and to obtain the best results, assuring the model stability, several simulations were performed to determine the ideal time step and horizontal viscosity to use in the model. For fixed horizontal viscosity and time step, different values of time step and horizontal viscosity were adopted, respectively, and the model results for the stations represented in Figure 1 were analysed.

The best model results are achieved with time step and horizontal viscosity of 2 minutes and 15 m^2^s^−1^, respectively. Several simulations were also performed to verify the model results independence relatively to the initial conditions, from which was concluded that the model spin-up period is about 48 hours. Consequently, these time step, horizontal viscosity and spin-up period values were included in all the study simulations.

The sea surface elevation (SSE) at the ocean open boundary results of the harmonic synthesis values that were determined for Cascais, using harmonic constants for this tidal gauge. Once the present study intends to analyse the astronomic tide propagation and the open boundary is very close to the tidal gauge position, this procedure minimizes the model errors since it as the advantage of remove other signals besides tides from the observed signal such as wind and variations of atmospheric pressure.

### Model Calibration

A mathematical model is by definition an attempt to approximate and reproduce real phenomena [33]. The approximations and parameterizations used for the model synthesis lead to discrepancies and deviations of model results from nature. Therefore, before being applied to a specific location, models should be calibrated and sometimes validated. Model calibration appears in various forms, depending on data availability, on water body characteristics, and most of all, on modellers' perceptions and opinions [34].

In this work, three distinct and complementary hydrodynamic model calibration methods are used, according to the methodology proposed by [22], and followed by [26], [35] and [36], in which an initial qualitative calibration is followed by the quantification of the models accuracy. The first step consists in a visual direct comparison between model results and observations, changing the model parameters in order to optimize their adjustment. In the second step, the root-mean square (RMS) errors and the Skill assessment between the SSE model results and observed data are calculated. The third step compares the SSE harmonic constants of the observed data and model results. Harmonic constants for 13 tidal gauges are available for the model calibration, providing an excellent coverage of the estuary (Fig. 1).

#### Comparison between predicted and observed series of SSE

Time series of SSE were synthesized for the 13 calibration stations applying the t_tide matlab® harmonic analysis package [37] to the available observed data (which consists in harmonic constants). It was reproduced the period from March 28 to May 1 of 2012. This period was also reproduced numerically, in order to obtain predicted time series for each one of the stations. Several simulations were performed, adjusting the Manning coefficient in order to optimize the adjustment between the model results and the observations. The parameter used in this adjustment during model calibration is the bottom stress represented in the Manning-Chézy formulation, being 0.04 the constant Manning coefficient value that induces model results that best fit the observations.

**Figure 2:**
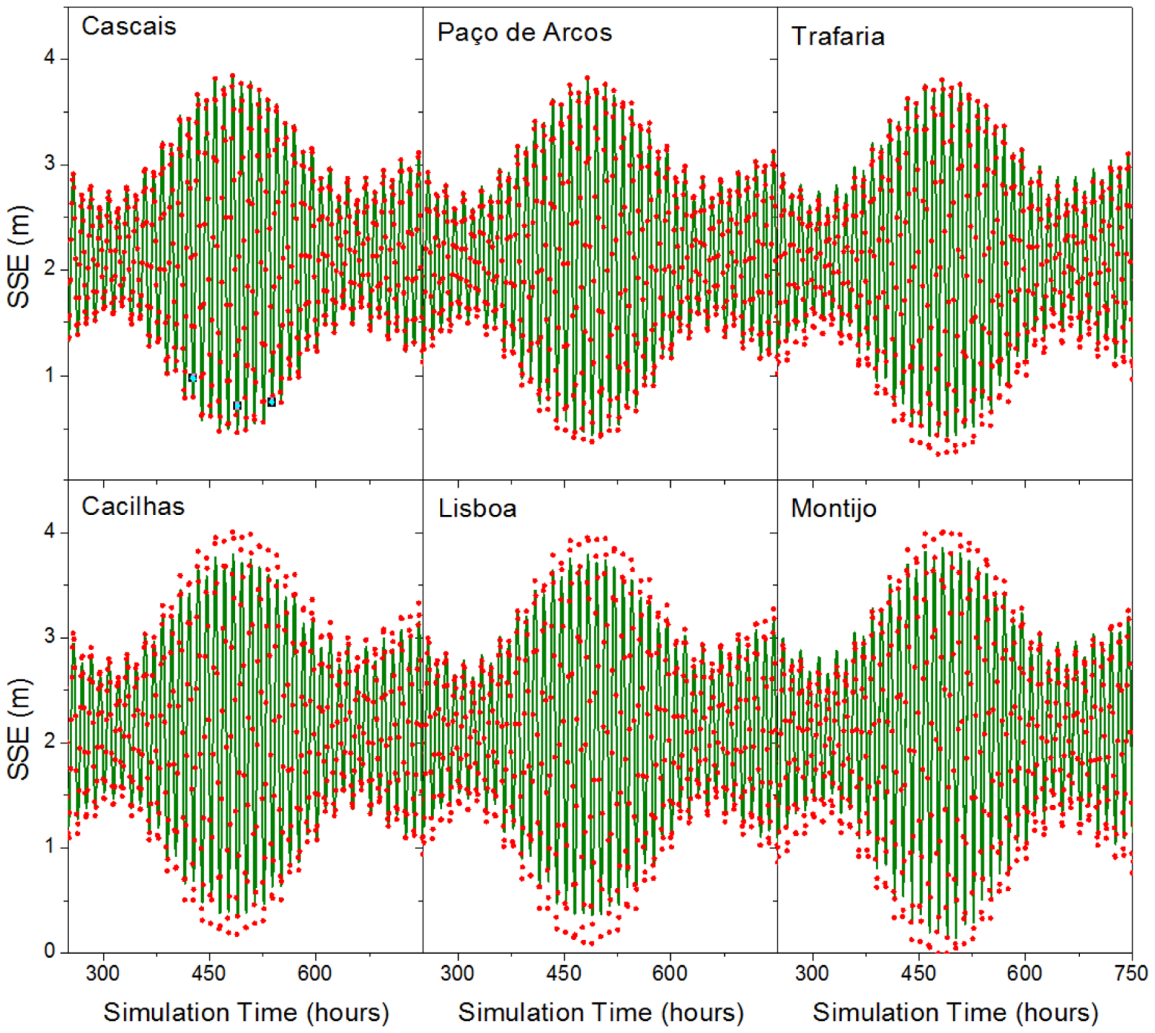
Comparison between the predicted (lines) and the observed (points) sea surface elevation time series for the stations of Cascais, Paço de Arcos, Trafaria, Cacilhas, Lisboa, and Montijo.

Figure 2 shows the comparison between the predicted (lines) and the observed (points) time series of SSE, for 6 of the stations used in the model calibration. The final adjustment between the series is good both in amplitude and phase although, during spring tides, sea surface elevation is underestimated by the model in some stations. The fortnightly tidal cycle is also well represented in the predictions, with a good reproduction of amplitude and phase, both in neap and in spring tides.

As the model open boundary condition is the SSE time series from Cascais, the agreement between the observed and predicted series for Cascais and Paço de Arcos is perfect, because these stations are very close to the ocean open boundary. The adoption of the observed SSE time series at Cascais as boundary condition allowed an optimization of the model results for the entire estuary, since obtaining a perfect match between predicted vs. observed time series at the lower estuary minimizes the errors for the remaining stations.

#### RMS errors and Skill assessment

The next step in the model calibration is the quantification of the model accuracy through the determination of the deviations between the model predictions and the observations. With this purpose were computed the root-mean square (RMS) errors and the Skill.

The RMS error of the difference between the predicted and observed SSE series was previously used by several authors (for example [15], [22] and [25]) to evaluate model's accuracy:
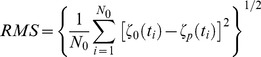
(1)where *ζ_o_(t)* and *ζ_p_(t)* are the observed and the predicted SSE, respectively, and *N_0_* is the number of measurements in the time series. According to [38], the RMS values should be compared with the local tidal range and if they are less than 5% of the local amplitude, the correspondence between the model results and observations is considered excellent. If these values vary between 5% and 10% of the local amplitude, the match is considered very good.

The model predictive Skill is being recently used to evaluate all prognostic quantities and is based on the quantitative agreement between model results and observations. This statistical method was developed by [39] and recently used by [38], [40] and [41].
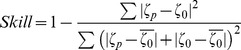
(2)


A Skill value equal to one corresponds to a perfect agreement between model results and observations while a complete disagreement yields a Skill of zero.

The RMS and Skill were computed for each calibration station and are presented in Table 1. The analyses of these results reveals that the model accurately reproduces the tidal wave propagation along Tagus estuary, since all the Skill values are very close to 1 and, likewise, RMS values are also between 0% and 15% of the local amplitude for almost stations.

**Table 1:** RMS and predictive skill for all the calibration stations.

Station	RMS (m)	SKILL
Cascais	0.015	1.000
Paço de Arcos	0.043	1.000
Trafaria	0.117	0.999
Cacilhas	0.119	0.999
Lisboa	0.148	0.999
Seixal	0.634	0.980
Alfeite	0.289	0.996
Montijo	0.255	0.997
Cabo do Ruivo	0.231	0.997
Alcochete	0.377	0.993
Ponta da Erva	0.705	0.974
**Póvoa de Sta. Iria**	0.358	0.994
**Vila Franca de Xira**	0.234	0.997

The poorer agreement was found for Seixal, Póvoa de Sta Iria, Ponta da Erva and Alcochete stations, because RMS and Skill values do not account the difference between the predicted and observed series in low tide. These stations are located in salt marsh zones, where the model simulates the wetting/drying along typical tidal cycle of tidal flats, with null SSE at the low tide. As the observed series were determined through harmonic synthesis, this effect is not observed in the data for these stations, and SSE values different from zero are predicted for low tide.

#### Harmonic analysis

**Figure 3:**
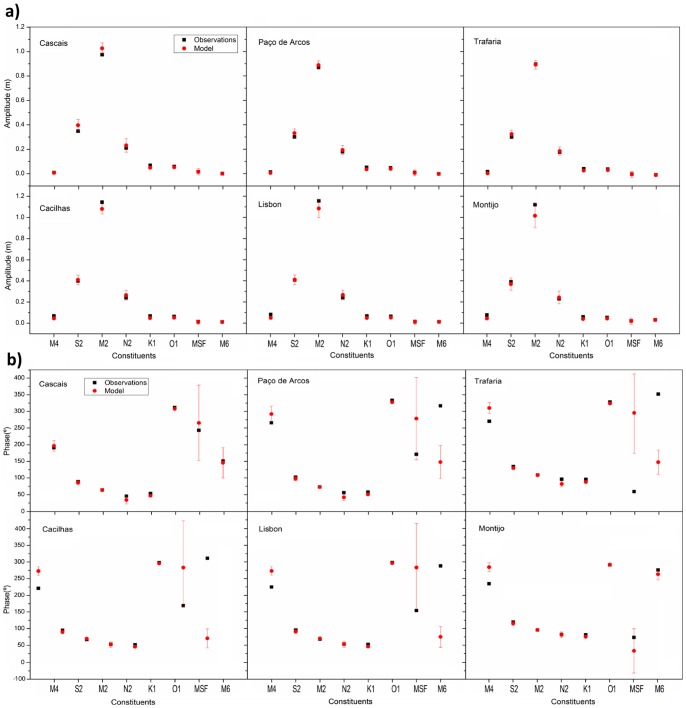
Comparison between the main harmonic constants amplitudes (a) and phases (b) determined from predicted (red) and the observed (black) sea surface elevation time series for the stations of Cascais, Paço de Arcos, Trafaria, Cacilhas, Lisboa, and Montijo. The values from predictions include error bars.

Comparative harmonic analysis of model predictions and observations is another method widely used to evaluate the accuracy of the model's performance ([22], [25], [26] and [35]). This method has the advantage of allowing the quantification of the models performance for each of the main tidal constituents, according to the tidal wave propagation characteristics along the estuary. In this study the harmonic analysis was performed using t_tide matlab® package [37] considering the harmonic constants values that were calculated from the model predictions for the 13 stations, which allowed the determination of the amplitude and phase of the main constituents, as well as the respective errors (Fig. 3). The period in analysis is the same that was used in the previous comparisons. Only the main harmonic constituents are presented, since the entire harmonic constituents list is extremely extensive. The predicted amplitudes interval for the semi-diurnal constituents (*M_2_*, *S_2_* and *N_2_*) includes the observed amplitudes at most stations, being *M_2_* the only exception for stations farther away from the estuary mouth (Ponta da Erva, Póvoa de Sta Iria and Vila Franca de Xira). For the phase, the results were good in all the stations, except a small deviation found for *N_2_* in Trafaria. For the diurnal constituents (*K_1_* and *O_1_*), the observed amplitudes met inside of the predicted interval for all the stations. For the diurnal phase the results were also very good since the only exceptions were found in Trafaria, Alfeite and Vila Franca de Xira (for *K_1_*) and in Póvoa de Sta Iria (for *O_1_*). For the lunisolar synodic fortnightly constituent *MS*_f_ the results were good in all stations both in phase and amplitude. For the shallow waters constituents (*M_4_* and *M_6_*) the predicted interval includes observed amplitudes in most stations, except in Lisboa, Montijo and Vila Franca de Xira for *M_4_*. Concerning the phase, the results were good only for Cascais and Paço de Arcos stations for *M_4_* and Cascais and Montijo for *M_6_*. In the remaining stations there was a great difference between observed and predicted phases, but it should be enhanced the great errors associated with the determination of the predicted phases for shallow water constituents.

The significant errors found for the shallow water constituents are not unexpected, as overtides are usually more difficult to predict correctly than the major astronomic constituents. *M_4_* and *M_6_* prediction errors may be due to bathymetric inaccuracies that cannot be corrected by the adjustment of the bottom friction coefficient. Even if these discrepancies are partially mitigated by the calibration for the major constituents, friction influences differently the non-linear constituents. However, as these constituents are generated through the advective and finite amplitude terms of the shallow water equations, which are found to be accurately predicted by the model, the results are expected to be precise enough to reproduce tidal asymmetry in this study.

#### Summary

The results show that the amplitude and phase of the tidal constituents can be considered satisfactorily represented by the model for the entire estuary. The deviations found between model predictions and observed data are not significant for most stations and constituents. Considering the direct comparison of the observed and predicted series of SSE, as well as the RMS and Skill values, it is verified that the best model results are found close to the estuary mouth but, generally, there is also a good adjustment for all the stations situated both in the central zone and far from the estuary mouth. The results were less satisfactory for stations close to the estuary margins, where the bathymetry is further complex (narrow channels and small bays) and the water is shallow (salt marsh and tidal flat areas). These higher discrepancies may be due to the inexact definition of the bathymetry (due its complexity, it is difficult to numerically define it) or to the natural difficulty in reproducing accurately the wetting/drying of these shallow areas.

It should be pointed out that the skill and RMS values are similar to those determined by [42] for the Guadiana estuary, [30] and [43] for the Patos lagoon, [40] for the Hudson River estuary, [22], [25], [26] and [35] for the Ria de Aveiro lagoon or [38] for Ria Formosa lagoon. The hydrodynamics of these estuarine environments was studied using finite elements and finite difference models. Those authors have found predictive skills ranging from 0.90 to 1 and RMS values of tidal elevations around 10% of the local amplitudes. As such, the results obtained show that the 2D application developed for Tagus estuary reproduces accurately the propagation of the tidal wave and thus the barotropic flows in this complex estuary. Therefore, it was concluded that this 2D model can be considered successfully calibrated and thus can be used to study important issues concerning the local variations in the estuary tidal regime.

## Tidal Characterization of Tagus Estuary

Due to the small dimensions and shallowness of Tagus estuary, its tidal properties are essentially driven by the oceanic tidal characteristics at its entrance and by changes and co-oscillation that results from tidal propagation along the estuary. Consequently, the analysis of changes in astronomical constituents along the estuary becomes essential to understand the factors ruling its tidal dynamics.

In the present work, the astronomical constituents *M_2_* and *O_1_* will be analysed because previous studies revealed that they are the major semidiurnal and diurnal constituents, which was also verified in the previous section results. *M_4_* is the most important shallow water constituent and has relevance in the definition of the local tidal asymmetry, and *M_6_* is important in the bottom friction effects analysis, and consequently will be also analysed.

The present section includes the results of the numerical simulations that were carried out in order to establish the tidal characteristics of Tagus estuary. The parameters defined during calibration of the model were kept and a time series of sea surface elevation (SSE) was applied for the period from October 8 (2011) to November 13 (2012) as ocean boundary condition. Hourly temporal estimation of SSE and horizontal fields of SEE throughout the computational domain were obtained. The time series of the velocity components were also computed at all grid points. The phase and amplitude distribution of the main harmonic *M_2_*, *O_1_*, *M_4_* and *M_6_* were determined and analysed based on those results by applying t_tide matlab® subroutines [37]. From this data set the form factor (requiring also the determination of amplitudes distribution for *S_2_* and *K_1_*), tidal ellipse, tidal asymmetry and tidal dissipation were also determined.

### Amplitudes and phase patterns of the main tidal constituents

As general pattern, it was observed that the variations in amplitude occur where changes in the estuary morphology and depth are larger, what happens in the transition zone between the main channel and in the initial and upper bay area. The results also reveal that the amplitude of the tidal diurnal constituent is one order of magnitude lower than the semi-diurnal in the whole system.

Concerning the amplitude of the higher harmonic constituent, *M_2_*, it increases progressively along the estuary until North of Cabo Ruivo (middle bay), diminishes until Póvoa de Sta. Iria, and raise again until Vila Franca de Xira (upper estuary). It ranges from 0.97 m at the mouth of the estuary to about 1.11 m near Vila Franca de Xira. The tidal amplification found at the middle bay is unexpected, since usually occurs a decrease in the amplitudes of the principal diurnal and semidiurnal constituents as tidal wave propagates along the most estuarine systems, due to friction and to non-linear transfers of energy [44]. However, as the tidal wave propagates into shallower waters within the estuary, it becomes distorted due to several physical processes, including reflection at the estuary head, which is responsible for standing-wave generation and local resonances [45]. Thus, the amplification found may be explained considering an estuarine resonance mode with a period close to twelve hours, due to the estuary length, which enhances the *M_2_* constituent as suggested by [16]. Following [46] and [47] quarter-wave resonance in a tidal basin occurs when:
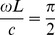
(3)where *L* is the length of the basin, 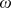 is the tidal period and *c* is the phase speed of the tide. For the *M_2_* constituent, 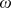 = 1.41
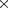
10^−4^ s^−1^ and the mean water depth determined from the numerical bathymetry developed for Tagus estuary in the frame of this study is 5.1 m, resulting in the phase speed, *c* = 7.07 ms^−1^. Thus, for quarter-wave tidal resonance, *L* = 78.76 km which is very close to the actual 80 km length of Tagus estuary. This result is therefore consistent with the tidal amplification found along the estuary for *M_2_* constituent.

Other authors have also reported this local resonance. According to [48], tidal amplitude inside the Tagus estuary is larger than at its mouth as a result of a small resonance effect and therefore he considered that this estuary can be hydrodynamically interpreted as a greater tidal basin connected to the ocean by a relatively straight and narrow channel. [15], for example, referred that tidal form number decreases along the system due to a resonant mode that increases the amplitudes of semidiurnal waves by roughly 40% in the upper estuary, leaving the diurnal waves mostly unchanged.

The tidal amplitude decline from Cabo Ruivo to Póvoa de Sta. Iria is due to the shallow depths of the upper bay that induce higher dissipation of tidal energy by bottom stress, therefore reducing the tidal amplitude. Upstream Póvoa de Sta. Iria the channel convergence increases again the tidal amplitude. In some narrow and shallow channels as Seixal and Ponta de Erva *M_2_* amplitude decreases to 0.50 m as result of the large bottom friction influence.

**Figure 4:**
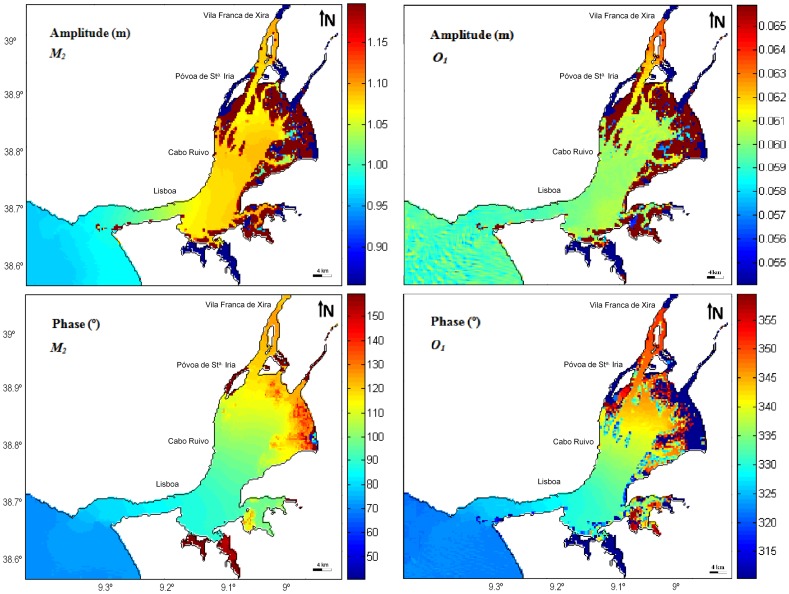
Semi-diurnal *M_2_* and diurnal *O_1_* constituents amplitude and phase for Tagus estuary.

Regarding to phase, differences in tidal propagation were also found along the two main areas of the estuary, the upper and lower estuary, with *M_2_* velocity being strongly attenuated in the shallow areas. In fact, it was observed a minor phase delay as tidal wave progresses toward the inshore zone of the system along the deepest lower estuary. It was also observed that the tide propagation delay is further significant in the bay due to the estuary morphology divergence and to depth decrease, which, in turn, makes bottom friction plus relevant inducing a lower tidal propagation velocity. Figure 4 shows that *M_2_* phase is ∼73° near the estuary mouth and ∼125° in Vila Franca de Xira, which means that the propagation delay for this constituent is about 1 hour and 47 minutes (52°). This value is slightly higher than that presented by [16], which refers to a delay of 1 h and 20 minutes.

The diurnal constituent *O_1_* has amplitude of ∼0.060 m near to the mouth, which keeps relatively constant along the estuary, reaching ∼0.064 m near to Vila Franca de Xira. Once again, in the channels mentioned above (Seixal, Ponta de Erva and Póvoa de Sta. Iria), the amplitude of the constituents drops to ∼0.050 m.

With respect to phase lag, *O_1_* shows a delay of about 1 hour and 50 minutes, which corresponds to a phase difference of 25°: near the mouth of the estuary is close to 325° and at the head is about 350° (Fig. 4).

### Amplitudes and phase patterns of the shallow water constituents

The sea surface water elevation (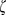) is usually a non-negligible fraction of the total water depth in estuarine shallow waters. As such, it has to be considered in the tidal wave celerity:
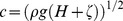
(4)where 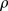 is water density, *g* the gravity acceleration and *H* the water depth. The wave crest travels rapidly than the troughs, resulting in shorter and further intense flood than ebb currents. Bottom friction also influences tidal wave propagation in shallow waters, delaying the water movement. As these processes depend on the square of current velocity, overtide generation is non-linear. The shallow water *M_4_* or *M_6_* constituents, both originated from *M_2_*, are therefore overtides that represent these distortions of the tidal levels normal harmonic variations, induced by shallow-water and friction effects. *M_4_* is essentially generated by the non-linear terms in the equations of continuity and momentum (advection), due to different celerity of crests and troughs, but friction can also play a minor role [49]. *M_6_* is basically generated by the quadratic friction term.

**Figure 5:**
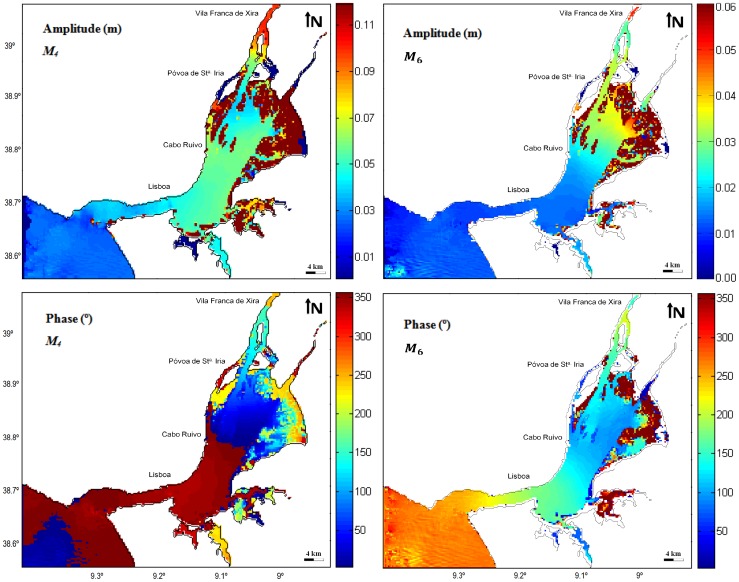
Shallow-water *M_4_* and *M_6_* constituents amplitude and phase for Tagus estuary.

The results for shallow water constituents demonstrate that their amplitudes are negligible in the coastal region, but increase rapidly inside the estuary (Fig. 5), being *M_4_* the most significant of these constituents. As a shallow water constituent, *M_4_* is strongly influenced by changes in system morphology and bathymetry. Therefore, amplitude and phase variations occur in areas where significant changes on system morphology and bathymetry arise. Figure 5 shows that *M_4_* systematically grows from Cascais to Cabo Ruivo, being the gradient higher between Cacilhas and Cabo Ruivo. From this point on, its amplitude decreases upstream towards Póvoa de Sta. Iria, and increases again with a strong gradient until Vila Franca de Xira. The amplitude varies from 0.02 near to the estuary mouth to 0.05 in the upper bay area, and close to Vila Franca de Xira the *M_4_* amplitude is ∼0.1 m. In the channel nearby Montijo it is observed a significant increase in amplitude from 0.06 m to 0.08, which can be explained by the shortening and shallowness features of this area. It is important to point out that in the shallow inner bay (between Lisboa and Vila Franca de Xira), *M_4_* has higher amplitude than the diurnal has.

The *M_6_* amplitude increases upstream, weakly until Cabo Ruivo and then with a stronger gradient toward Póvoa de Sta.Iria, where it reaches its maximum values. In the main channel, the amplitude is relatively constant, approximately 0.013 m. However, from the initial area of the bay to the upper bay, is observed an amplitude increase of ∼0.018 m: the amplitude varies from ∼0.014 to ∼0.032 m, respectively. Near Vila Franca de Xira, Figure 5 shows a decrease of ∼0.005 m, which means that in this area the amplitude value is about ∼0.027 m.

As Cabo Ruivo and Póvoa de Sta. Iria seem to be turning points for the shallow-water constituents, this might correspond to changes in the respective generating processes. [16] also reported this fact, stressing the dominance of advective accelerations (responsible for *M_4_* generation) in the deeper part of the estuary compared with the larger influence of friction (responsible for *M_6_* generation) in the shallower part of the estuary (Fig. 1).

Regarding to the *M_4_* phase lag, in the initial zone of the estuary, the propagation delay is very small: from the mouth to the upper bay area, the phase difference is about 15°. However, this delay increases upstream and the difference becomes more significant near Vila Franca de Xira (varies from ∼60° to ∼210°, which corresponds to 2 hours and 30 minutes) as a result of strong irregularities in the morphology. Concerning to *M_6_* phase, the lower values (∼75°) are observed at the upper bay, characterized by the shallower depths, which induce the higher bottom friction and where the *M_6_* higher amplitudes were found. As a result it should be concluded that this constituent is essentially generated in this area, propagating up and downstream along the estuary. The phase increases to ∼114° at the end of the main channel and to ∼160° at the mouth, and to ∼124° near Vila Franca de Xira.

### Form factor

The relative importance of diurnal and semi-diurnal constituents could be expressed in terms of a form factor or number factor. In this study, the form factor for the entire estuary was assessed, derived from the harmonic analysis results and applying the following expression [50]:
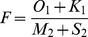
(5)where *O_1_*, *K_1_*, *M_2_* and *S_2_* are the amplitudes in meters of correspondent constituents. Tide is semidiurnal if 0<*F*<0.25; mixed tide – semidiurnal tides predominate if 0.25<*F*<1.5; mixed tide – diurnal tides predominate if 1.5<*F*<3.0; and, finally, the tide is diurnal if *F*>3.0 [45].

**Figure 6:**
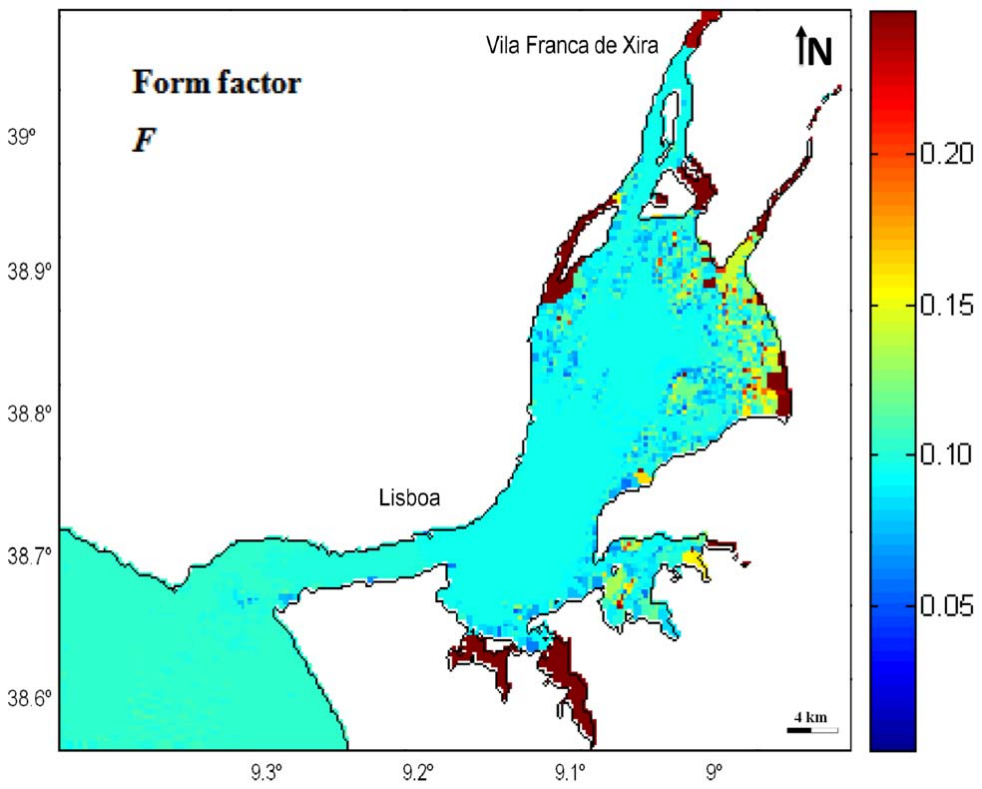
Form factor distribution in the Tagus estuary.

As this estuary is mesotidal and tides are primary semi-diurnal, *M_2_* and *S_2_* amplitudes are significantly higher than those of diurnal constituents *O_1_* and *K_1_*, being thus expected a low form factor *F*. In fact, Figure 6 demonstrates that *F* value is less than 0.25 throughout the estuary, meaning that tide is semidiurnal. In fact, *F* is approximately constant and equal to 0.1 within the system. Although almost imperceptive due to the colour scale that was necessary to applied to represent the model results, it is verified a slightly decrease of the form factor along the system, mainly within bay, mostly as a result of *M_2_* increased amplitude, as earlier discussed. Tidal form number decrement along the system was described by other authors, such as [15], referring that this pattern results from the resonant mode that increases the semi-diurnal waves amplitudes inside the estuary. In shallowest areas such as in the upper bay margins and mostly in channels along the Seixal and Póvoa de Sta. Iria, form factor increases, approaching 0.49, due to the strong decrease of the semi-diurnal constituents amplitudes in these areas, as was previous noted.

### Tidal Ellipses

**Figure 7:**
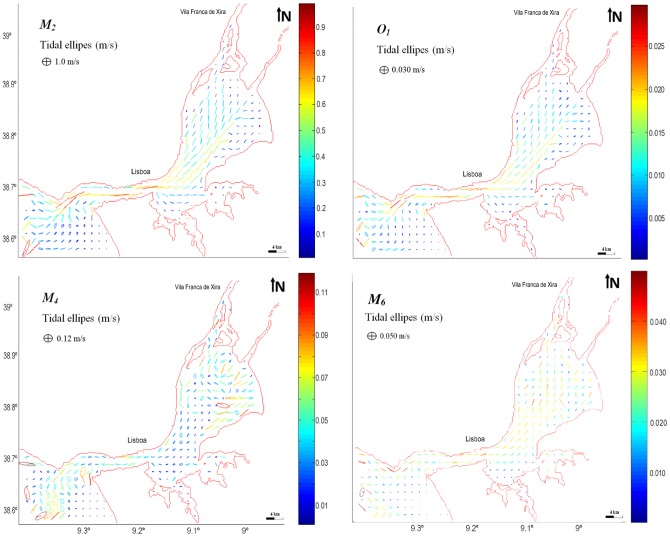
Tidal ellipses distribution of tidal constituents *M_2_*, *O_1_*, *M_4_* and *M_6_* in Tagus estuary.

The determination of tide ellipses for the main tidal constituents allows the assessment of tidal currents patterns along the estuary [31]. Figure 7 shows the tidal ellipses for semi-diurnal, diurnal and shallow water constituents in the Tagus estuary, and from its analysis is found that in general all ellipses have high eccentricity, meaning that tidal currents are mainly rectilinear and the ellipses sense of rotation should not be significant. This pattern is induced by the constraints imposed by local bottom topography and geometry. Therefore, the ellipses orientation (for semidiurnal, diurnal and *M_6_* constituents is similar) reveals the importance of these factors on tidal fluxes, with main flux occurring essentially through deeper zones. In fact, tidal ellipses results revealed preferential flow channels in the bay, with highest speeds being reached in two deepest areas. From Figure 7 it is also observed that in the Tagus estuary currents attain maximum values at the system mouth (∼0.8 for the *M_2_* constituent; ∼0.035 for *O_1_* constituent). The general patterns found for both constituents show that current velocity decreases from the lower to the upper estuary. Lower current values were found in the bay, near to land boundaries. In the upper zone of the bay, the current loses intensity and values decrease, approaching 0.3 m/s in the case of *M_2_* and approximately 0.02 m/s for *O_1_*.

In turn, *M_4_* shows a different pattern. As a shallow water constituent, the highest values are found when significant differences in morphology and bathymetry arise (upper zone of the bay, essentially), being ∼0.08 m/s. In the main channel, velocity is about 0.04 m/s and in the initial zone of the bay its values decrease to 0.02 m/s. It should be referred that *M_4_* current intensity is locally important, since it is often larger that *O_1_* intensity, making this contribution the 4^th^ most important in Tagus tidal dynamics. The pattern found for *M_6_* is similar to those found for semi-diurnal and diurnal constituents, although with very low current intensities.

### Tidal Asymmetry

A symmetrical tide occurs when the tide rise and fall have identical duration, presenting approximately equal maximum velocities, which results in no net overall sediment transport. Tidal asymmetry happens when the ebb and flood durations are unequal and it is caused by tidal wave distortion during propagation into shallow waters, along the coastal shelf and on entry into estuaries. This phenomenon is induced by nonlinear effect mechanism of tidal propagation, resulting from finite amplitude effects on the non-linear advection and friction terms. Likewise, information of tidal asymmetry may become very useful to determine currents patterns, including the current flood or ebb prevalence [51]. In many coastal worldwide areas where tide is semi-diurnal, as in the Tagus estuary, the dominant tidal constituent is *M_2_* and thus the most significant shallow water constituent is *M_4_*, the first harmonic of *M_2_*. However, as observed in section 4.2, *M_6_* should also be considered in the case of Tagus estuary. Tidal distortion (asymmetry) can be represented by harmonics of astronomical tidal constituents [44]. Thus, the ratios of *M_4_* and *M_2_* and of *M_6_* and *M_2_* amplitude (which corresponds to the *A_r_* values) can be analysed to determinate the tidal asymmetry magnitude generated within the Tagus estuary. Similarly, relative phases of *M_2_* and *M_4_* and of *M_2_* and *M_6_* determine the type of asymmetry. Therefore, the asymmetrical coefficients *A_r_* (amplitude ratio) and 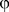 (relative phase) can be represented by the following equations:
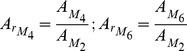
(6)

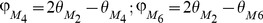
(7)where *A* is the amplitude, 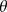 indicates the phase and *M_6_*, *M_4_* and *M_2_* correspond to the tidal constituents. The flow is *flood dominant* if 0°<
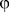
<180° and *ebb dominant* if 180°<
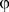
<360°. Eventually, this creates net sediment transport over a tidal cycle, with *flood dominated* currents resulting in net sediment transport into the estuary and, in the other side, under *ebb dominant* conditions, the opposite happens and net seaward transport will take place causing sediment export from the estuary. In fact, tidal asymmetry is frequently the dominant factor in causing net sediment transport and deposition which results in sediment trapping in coastal areas and estuaries [52]. The navigability of estuarine channels and estuaries geological evolution are consequently affected by tidal asymmetry [44] which is, therefore, a fundamental factor for morphological development of tidal basins.

#### Amplitude ratio and relative phase difference: M_4_

**Figure 8:**
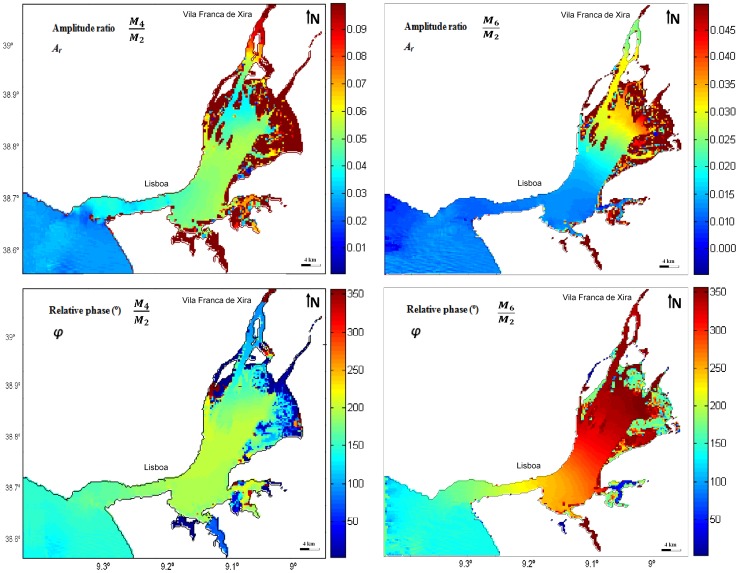
Amplitude ratio (*A_r_*) and relative phase (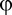) for the *M_4_* and *M_6_* constituents in Tagus estuary.

The analysis of Figure 8 reveals that there is a progressive increase of *A_r_* along the estuary. Entering in the system, tide suffers a severe distortion as a result of the strong shortening that is felt in the input channel (lower estuary): from the ocean to the initial zone of the channel, amplitude ratio increases from 0.025 to 0.041, approximately. At the bay, where depth is less, *A_r_* value exceeds 0.048. The dark colours areas (dark brown) northeast from the bay correspond to narrow and shallow channels and tidal flats, which promote energy dissipation due to the bottom friction effects and, consequently, represent areas with strong tidal distortion. Some deeper channels that do not uncover with the tide, as the Seixal and Póvoa de Sta. Iria channels, present maximal asymmetry values, close to 0.549, as a result of the water velocity increment due to the channel convergence.

Throughout the system, the amplitude ratio *A_r_* is always higher than 0.02 (excluding the mouth of the estuary), which allows to state that tidal asymmetry occurs in the whole system. An asymmetric Tagus estuary behaviour is also mentioned by [3].

In some other coastal systems, such as the Ria Formosa [53], there are areas where amplitude ratio value is zero, so it does not occur tidal asymmetry, and others that reach values of 0.9. Given this example, is verified that tidal asymmetry in the Tagus estuary is relatively low, with values of *A_r_* higher than 0.9 only in the narrower and shallower channels (as near Seixal and Vila Franca de Xira).

**Figure 9:**
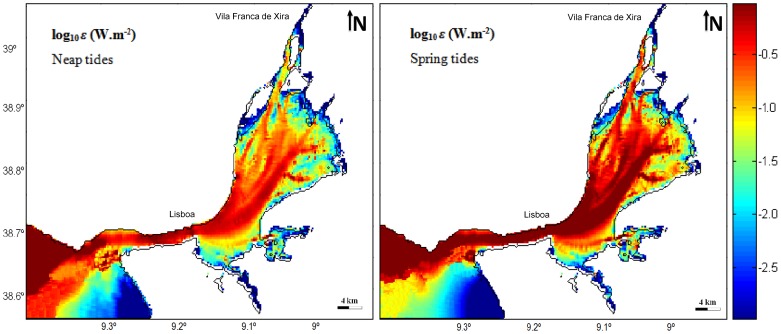
Spatial-logarithmic distribution of the average dissipation of tidal energy in neap and spring tides in Tagus estuary.

For relative phase is concluded that results are in agreement with bibliography that state that this estuary is dominated by the ebb, which means that rising tides duration exceeds those of falling tides, causing a tendency for stronger ebb than flood tidal currents. Actually, Figure 8 shows that for the entire estuary (excluding narrower and shallower channels) relative phase value is over than 180°, indicating *ebb dominance*. Relative phase values are maximum in mid-estuarine region (transition between the inlet channel and the inner bay) revealing that the highest distortion on water level variation towards *ebb dominance* occurs in this area. The *ebb dominance* has a strong impact on sediment dynamics, promoting sediment transport out of the estuary and therefore inducing erosion in these areas. However, in narrower and shallower channels relative phase differs from the rest of the estuary, similarity to the pattern observed for the amplitude ratio: relative phase is less than 180° (Seixal, Ponta de Erva and Vila Franca de Xira channels – Figure 9). Thus, *flood dominance* occurs, favouring sedimentation processes in these places with consequent accretion.

#### Amplitude ratio and relative phase difference: M_6_

The majority of studies on tidal asymmetry only analyse the importance of *M_4_* on the water level distortion, thus only a small attention has been devoted to research *M_6_* contribution to tidal asymmetry. In this work, amplitude ratios *AM_6_/AM_2_* and relative phase 3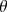 *M_2_*-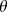 *M_6_* were also determined from the harmonic analysis results, in order to identify a possible relation between these parameters and tidal asymmetry. Results for water level time series are presented in Figure 8, showing a progressive increase of *A_r_* along the estuary. Although *A_r_* values are approximately constant in the main channel (about 0.013), at the bay (where the depth is lower comparatively to the main channel) values are significantly higher. In fact, there is an increase of 0.019 within the bay: *A_r_* rises from 0.013 to 0.033 from the initial to the upper bay. The large increase of *A_r_* along the bay reveals the intensification of the relative importance of bottom friction, resulting in a low water delay [49], and therefore contributing to the decline of the time between low and high water. Thus, *A_r_* pattern at the upper bay can be probably related to *flood dominance* trend, previously identified though relative *phase 2
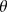
M_2_-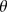
M_4_*. *A_r_* amplitude ratio is always higher than 0.01 throughout the system, revealing that tidal asymmetry occurs in the whole estuary.

For relative phase, excluding the initial zone of the main estuary where relative phase values of 152° are observed, values are always higher than 180° in the whole estuary. At the end of the main channel the relative phase values are about 190° and in the upper bay reach 342°, which means a difference of 152° along the bay. Close to Vila Franca de Xira relative phase values decrease to 338°.

### Tidal Dissipation

In this study the mean rate dissipation of tidal energy per unit area due to bottom friction is estimated from the application of the following equation [47]:
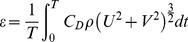
(8)where *C_D_* is the bottom friction used in the calibration of the model and *U* and *V* are the *x* and *y* components of the current, respectively. The integration presented in equation (8) was taken over one tidal cycle for neap and spring tide cases in this study.

Figure 9 shows spatial-logarithmic distribution of average tidal energy dissipation in neap and spring tides in the Tagus estuary. Energy dissipation values were analysed through *ε* logarithm for a better interpretation of results.

According to [7], values of the average rate of energy dissipation arise, in general, associated with areas where boundary friction is high or where a sudden change of estuary geometry occurs. Thus, changes in estuary bathymetry and geometry could have a substantial effect in the way that tidal wave energy is dissipated and may even be responsible for tidal wave distortion. From Figure 9 is observed that the highest values are reached in the transition from the ocean to the input channel, in the main channel (lower estuary) and in the central deepest area of the bay (upper estuary) (Fig. 1). During neap tides, the value of maximal dissipation arises near the estuary mouth, in the narrowest part of the main channel and in the deepest area of the bay where depth varies between 0 and 12 meters. Without considering the wetting/drying areas, the remaining places in the estuary where maximal dissipation is found show that dissipation decreases with decreasing depth: at higher depths dissipation is close to 0.5 W/m^2^ and decreases progressively with depth, reaching values of ∼0.2 W/m^2^.

During spring tides, in the transition zone, in the main channel and in the central area of the bay, the maximal dissipation is ∼1 W/m^2^. Indeed, at these locations, dissipation is high and almost constant, except in the broad area of the channel where, along the shores, the dissipation is lower. In the bay, the energy dissipation decreases to ∼0.03 W/m^2^ in shallower channels (depths between 0 and 2 meters). The closer shore areas corresponding to wetting/drying zones have lower values. In the entrance channel dissipation is maximal, which can be justified by the considerable depths in the central area (turning the bottom friction negligible) and by the channel convergence and shortening (favours an increase in current velocity). Likewise, as the channel is narrow and the depth decreases along its banks, the boundary friction can also foster an increase in energy dissipation. In the bay, there is a strong depth decline comparatively to the entrance channel and a significant decline as well from the central area to the peripheral areas, resulting in a speed decrease in these areas. As in main channel, the central area of the bay is also a place where currents are strongest relatively to the peripheral areas that have shallower depths, which suggests that dissipation is associated with high current velocities. In areas of minor depth a decreased flow occurs and dissipation is actually smaller. Moreover, since the dissipation is apparently related to high current speeds, looking at Figure 9 and taking into account the maximum dissipation, it can be also concluded that flows occurs through preferential channels (as mentioned above), where depths are greater.

The results show that tidal dissipation is higher during spring tides once tidal currents are weaker in neap tides, inducing less energy dissipation. In general, the maximum values of dissipation for neap tides are close to 0.5 W/m^2^ while for spring tides they are ∼1 W/m^2^, showing that maximal dissipation during neap tides is about half of the maximum that occurs in spring tides. This pattern is in accordance with the results obtained from other coastal systems such as the Ria Formosa [53], Shark Bay [47], Maputo Bay [54] and Ria de Aveiro [55], where the tidal dissipation calculated for neap tides is considerable lower than for spring tides. Comparatively to Ria Formosa and Shark Bay, the results suggest that in general Tagus estuary represents a similar potential of energy dissipation once the maximum values in those coastal systems are approximately 1 and 0.7 W/m^2^. Maputo bay presents values of ∼0.1 W/m^2^ over a large proportion of the bay although it also could reaches maximum values of 1 W/m^2^ while it was identified maximum values of energy dissipation of ∼1.7 W/m^2^ in Ria de Aveiro lagoon.

The results of the present study are in agreement with that observed for the other studied parameters: Tagus estuary bathymetry, coastline features and bottom friction have an extremely important role in the circulation patterns of this system. Previously authors refer this fact and, for example, [3] says that the Tagus estuary bottom topography is the main constraint to the tidal currents direction.

## Conclusions

A two dimensional numerical model has been used to examine the tidal dynamics of Tagus estuary. The first step of this study revealed that the model accurately reproduces the tidal wave propagation in this estuary, so it can be considered successfully calibrated. Therefore, the tidal dynamics of Tagus estuary has been successfully resolved through the application of the SIMSYS2D model. It should be pointed out that the use of a 2D model assures a good reproduction of the estuarine tidal dynamics, even neglecting the 3D baroclinic effects. Model results indicate that estuary bathymetry and coastline features, namely the differences in morphology and depth from the lower to the upper estuary, play a key role in setting the circulation patterns found in the system. This influence is observed in major and shallow water tidal constituents, tidal asymmetry, tidal dissipation and tidal ellipses patterns.

Results of amplitude and form factor have given evidence that tide is semidiurnal. The *M_2_* amplitude increases within the estuary from Cascais to Ponta da Erva. Even though losing energy to the shallow-water constituents, the semidiurnal tide grows up as it propagates from the mouth until Ponta da Erva, decreasing further upstream at Vila Franca de Xira. This pattern suggests that between Ponta da Erva and Vila Franca de Xira there is a fraction of the tidal energy reflected somewhere, which is consistent with the existence of a resonant mode with the *M_2_* period.

The results also showed differences in the tide propagation in two main areas of the estuary (the upper and lower estuary), resulting from a balance between convergence/divergence and bottom friction and advective effects. There is a phase delay as the tidal wave moves toward the inshore zone of the system. In the shallow areas the *M_2_* is strongly attenuated, while the opposite occurs for the *M_4_* and *M_6_*, which are strongly amplified, thus inducing tidal asymmetry. The amplitudes of both shallow-water constituents *M_4_* and *M_6_* enlarge along the estuary, from the mouth to the head. The *M_4_* constituent amplitude increases from Cascais to Cabo Ruivo, whereas *M_6_* amplitude is smoothly enlarged from the mouth to Cabo Ruivo, and with a higher gradient until de estuary head. Considering that the *M_4_* generation is essentially due to the nonlinear effects of advection and that *M_6_* is mainly induced by bottom friction, these patterns suggest that the advective accelerations are dominant from the estuary mouth to Cabo Ruivo, while bottom friction takes a larger significance upstream that zone.

The ellipses orientation reveals the importance of the bottom topography and of the bay geometry on the tidal fluxes, with the main flux through the deeper zones. Tidal asymmetry parameters indicate *ebb dominance* for most of the estuary, so there is a general trend for a sediment exportation, and consequently for erosion with the natural deepening of these areas. The results for the tidal dissipation showed that the dissipation is higher in deeper areas where tidal currents are stronger, which is the case of the main channel (upper estuary) and in two preferential channels of the bay. The analysis of this parameter simultaneously with tidal ellipses orientation show that the flow in the Tagus estuary occurs by preferential channels, where current speeds are higher due to their highest depths.
